# Post-activation Potentiation: Effects of Different Conditioning Intensities on Measures of Physical Fitness in Male Young Professional Soccer Players

**DOI:** 10.3389/fpsyg.2019.01167

**Published:** 2019-06-06

**Authors:** Cristina Petisco, Rodrigo Ramirez-Campillo, Daniel Hernández, Oliver Gonzalo-Skok, Fabio Y. Nakamura, Javier Sanchez-Sanchez

**Affiliations:** ^1^Research Group Planning and Assessment of Training and Athletic Performance, Pontifical University of Salamanca, Salamanca, Spain; ^2^Laboratory of Human Performance, Research Nucleus in Health, Physical Activity and Sport, Department of Physical Activity Sciences, Universidad de Los Lagos, Osorno, Chile; ^3^Faculty of Health Sciences, University of San Jorge, Zaragoza, Spain; ^4^College of Healthcare Sciences, James Cook University, Townsville, QLD, Australia; ^5^Department of Medicine and Aging Sciences, “G. d’Annunzio” University of Chieti-Pescara, Chieti, Italy

**Keywords:** plyometric, speed, fatigue, warm-up, soccer

## Abstract

The aim of this study was to compare the effects of different warm-up conditioning intensities on the physical fitness (i.e., post-activation potentiation -PAP), of professional male field soccer players. Athletes (*n* = 10; age: 21.6 ± 3.2 years) completed a control warm-up and warm-ups aimed to induce PAP, in random and counterbalanced order. After control and experimental warm-up sessions participants completed a triple hop test with the dominant (H3Jd) and a non-dominant (H3Jnd) leg, a squat jump (SJ), a countermovement jump (CMJ), a change of direction ability (COD) test, a repeated sprint with a COD (RSCOD) test and a linear 30-m sprint test (S-30). The control warm-up (WU) protocol was designed according to athlete’s regular warm-up practice. The experimental warm-ups included the same exercises as the WU, with addition of one set of half-back squats for 10 repetitions at 60%, 5 repetitions at 80%, and 1 repetition at 100% of 1RM (60%-1RM, 80%-1RM and 100%-1RM, respectively.) Threshold values for Cohen’s effect sizes (ES) were calculated and used for group’s comparison. Likely to most likely improvements were shown in H3Jd (*ES* = 0.52), H3Jnd (*ES* = 0.51), COD (*ES* = 0.38), fasted sprint (RSCODb) (*ES* = 0.58) and the total time of all sprints (RSCODt) (*ES* = 0.99) only after the 80%-1RM protocol in comparison to the WU. Conversely, 100%-1RM and 60%-1RM protocols, compared to WU, induced possibly to most likely poorer performance in all jumps, COD and RSCODb (*ES* = −0.07 to −1.03 and *ES* = −0.48 to −0.91, respectively). Possibly to most likely improvements were shown in all jumps, COD, RSCODb and RSCODt after the 80%-1RM warm-up protocol in comparison to the 100%-1RM and 60%-1RM warm-up protocols (*ES* = 0.35 to 2.15 and *ES* = 0.61 to 1.46, respectively). A moderate warm-up intensity (i.e., 80%-1RM back squat) may induce greater PAP, including improvements in jumping, repeated and non-repeated change of direction speed in male soccer players.

## Introduction

Aside from the total distance covered, performing high-intensity actions repeatedly during a match is a key feature of soccer (Stølen et al., 2005). During a competitive match, soccer players may perform ∼1400 short-duration maximal or near maximal intensity activities, including sprints, change of directions (COD), tackling, accelerations, decelerations, jumps, among others (Iaia et al., 2009). Although training programs may improve such performance actions at long-term, short-term (or acute) improvements may also be induced by warm-up activities, a method routinely used by athletes, coaches and strength and conditioning specialists to improve muscle force and power involved in athletic performance during competition (Evetovich et al., 2015).

A large amount of research regarding the effects of a warm-up on human performance have been conducted (Rahimi, 2014; Barnes et al., 2015; González-Mohíno et al., 2018). There is a general consensus pointing the benefits of a warm-up on subsequent performance (Lockie et al., 2017). However, the optimal warm-up strategy for soccer players before a match is not well established (Hammami et al., 2018). Most soccer-related warm-up strategies involved static and dynamic stretching, neuromuscular activities, and short-duration high-intensity activities (Zois et al., 2011). Regarding the latter, they may induce post-activation potentiation (PAP) (Evetovich et al., 2015).

The PAP is a phenomenon in which neuromuscular performance characteristics are enhanced after intense contractile stimulation (Hodgson et al., 2005). The PAP in humans may be induced by isometric maximum voluntary contraction (Hamada et al., 2000), high-intensity resistance stimulus (McBride et al., 2005), and plyometric exercise (Turner et al., 2015). Although the existing research tends to reveal inconsistent findings (Moir et al., 2009), some studies has shown that performing muscular contractions under near-maximal load conditions improves subsequent performance during movements requiring large muscular power outputs of the stimulated muscle groups (Hodgson et al., 2005). However, there is little research regarding the effects of heavy resistance exercise on subsequent performance in soccer (Hammami et al., 2018). Some authors indicated that heavy resistance exercise may improve subsequent jump and COD (Zois et al., 2011), repeated sprints (Low et al., 2015; Sanchez-Sanchez et al., 2018) and single linear sprint (McBride et al., 2005; Tillin and Cooke, 2009).

The exact mechanism responsible for this PAP response remains uncertain (Lockie et al., 2017). Chemical, neuromuscular and mechanical changes may occur that temporarily aid the contractile properties of muscle tissue (Sale, 2004). One common PAP mechanism theory indicates the phosphorylation of myosin light chains resulting from the initial muscle activity, which would turn the actin and myosin molecules more sensitive to calcium (Ca2+) availability (Tillin and Bishop, 2009). Also it is speculated that PAP can increase the excitability of motoneurons (Hodgson et al., 2005) and enhancement of neural output by recruitment of faster motor units (Hamada et al., 2000; Sale, 2004). Moreover, it was also reported that pre-loading may increase muscle stiffness (Moir et al., 2009). Previous studies have suggested that PAP responses might be dependent on individual characteristics (Sanchez-Sanchez et al., 2018). For instance, stronger subjects exhibited a greater PAP response when compared with weaker counterparts (Seitz and Haff, 2016). In contrast, other studies concluded that performance after a PAP inducing activity was not related to training status (McBride et al., 2005). Furthermore, it has been proposed that the PAP may be related to the type of muscle fiber being activated (Wilson et al., 2013), with a higher proportion of fast fibers being related to greater PAP effect (Sole et al., 2013).

Therefore, in order to maximize PAP, these factors (e.g., training status) should be taken into account together with the load used during the warm-up (Lockie et al., 2017). McBride et al. (2005), for example, demonstrated that 1 set of 3 repetitions at 90% of one repetition maximum (1RM) significantly improve sprint time performance, whilst 1 set of 3 repetitions at 30%-1RM did not. In the same context, a previous study conducted with male soccer players showed that in order to induce optimal running speed enhancements, it is necessary to set the intensity of the warm-up protocol with loads ≥80%-1RM (Rahimi, 2014).

The competition rules require the physical trainers to finish the warm-up 15–20 min before the start to the matches. The PAP could be used between the end of the warm up and the start of the game, to maintain the level of activation in the players (Russell et al., 2014). Although greater loads have been recommended to induce PAP in strength and power tasks (McBride et al., 2005; Rahimi, 2014), its practical application in soccer is difficult. This is because of the limited time-frame separating the end of the warm-up and the start of a soccer match which is not sufficient to include multiple sets (Russell et al., 2014). Therefore, the aim of this study was to compare the effects of different warm-up conditioning intensities on physical fitness (i.e., PAP), of professional male field soccer players.

## Materials and Methods

### Participants

Professional male soccer players (*n* = 10; age: 21.6 ± 3.2 years, body height: 177.9 ± 4.3 cm, and body mass: 69.5 ± 3.1 kg) with ≥6-years of training and competition experience were recruited for the study. Their regular training schedule involved four training sessions plus a competitive match per week in the Spanish second division “B.” All participants: (1) were field players (four defenders, four midfielders and two forward), (2) were free of injuries in the last 3-months, (3) had regularly trained and competed in the past 6-months and (4) haven’t got any lower extremity surgery in the past 2-years. Soccer players signed an informed consent before starting the data collection. The protocol was approved by the Ethics Committee of the Pontifical University of Salamanca (Annex III, Act 13/2/2019) and conformed to the latest version of the Declaration of Helsinki.

### Procedures

The experiments were conducted during the competitive period of the season 2018. The control and experimental warm-up sessions were completed in a random, counterbalanced order, completed in a period of 3-weeks. The tests during control and experimental sessions were completed in the same order, between 15:00 and 20:00 h, at an indoor venue, with the same sports clothes and by the same investigator, who was blinded to the group allocation of the participants. To avoid the effects of fatigue on testing results, participants completed the control and the experimental warm-up sessions no less than 48 h after the last training/competition session. Each intervention was applied twice, making the tests in the following order. After control and experimental warm-up sessions participants completed measure in day 1: triple hop test with dominant (H3Jd) and non-dominant (H3Jnd) leg, squat jump (SJ) and repeated sprint with COD (RSCOD); measure in day 2: linear 30-m sprint test (S-30), countermovement jump (CMJ), COD test. The recovery between tests was 1 min. Participants were asked to attend each session under an adequate feeding and hydration state. The testing protocols were performed in the facilities where athletes usually train and compete.

#### Familiarization and Maximal Dynamic Strength Test

During a 120 min familiarization session, athletes simulated the warm-up protocols and completed a maximal dynamic strength test (1RM) in order to assess the specific loads to be used during PAP warm-up sessions. The maximal dynamic strength was assessed through the half back squat exercise using the Smith machine (MultipowerPeroga^®^, Murcia, Spain), with the barbell constrained to move along the vertical axis. The 1RM test was preceded by a 5-minutes low-intensity run in which the heart rate not exceed at 140 b⋅min^−1^ (Polar RS800CX, Electro Oy, Kempele, Finland), and by 5 and 3 half back squat repetitions at an estimated 50% and 70% 1RM, respectively. In the initial position the barbell was at shoulder-level, feet at shoulder-width distance, and knee and hips in full extension. Adhesive marks were added to the floor and the barbell to assure consistency in the hands and feet position during testing. In addition, a wooden seat with adjustable heights was placed behind the subjects to keep bar displacement and knee angle (∼90° knee angle) constant on each half back squat attempt. The 1RM load was defined as the maximum weight that could be lifted once using the proper exercise technique through a full range of motion (Okuno et al., 2013). A 3-minutes rest interval was adopted between attempts, and the subjects had up to five attempts to obtain their 1RM.

### Jumping Test

Athletes completed the H3Jd and H3Jnd (Noyes et al., 1991). Participants take maximal jumps forward as far as possible on the testing leg and land on two legs during the final jump. At the end of each horizontal jump attempt, athletes maintained the landing position for a brief moment. Soccer players also completed the CMJ and SJ tests following previous suggestions (Maulder et al., 2015), with minimal flexion of the trunk during take-off. Jumping was measured with a contact mat (Globus Ergo System^®^, Codogné, Italy). In all jumps, the hands were used freely, except during the SJ and CMJ, where athletes positioned arms akimbo. Athletes performed two maximal trials for each test with 1-minute of rest in between. The best value achieved was selected for analysis.

### Change of Direction Ability Test

**FIGURE 1:**
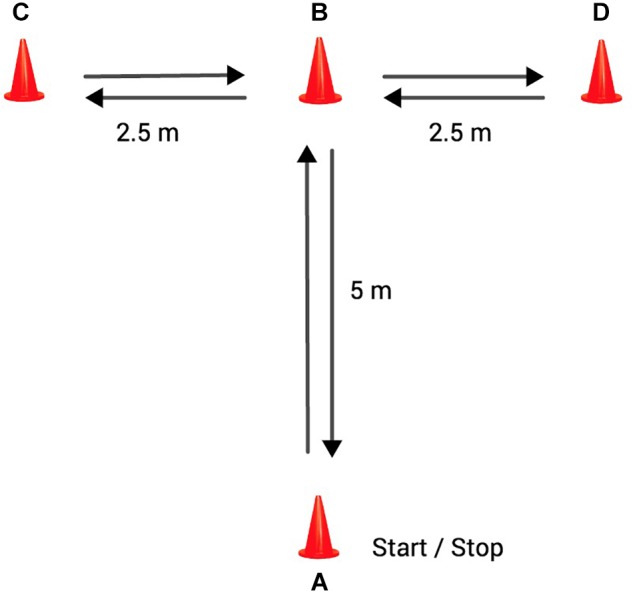
Change of direction ability test.

Athletes also completed a modified *t*-test (Sassi et al., 2009) to evaluate COD. A photocell gate system (Witty, Microgate^®^, Italy) was used to record the time. The players performed the test using the same directives as the traditional test, although they were not required to move laterally or face forward (Figure 1). The players had to touch the top of the cones instead of its base. The displacement followed this route: AB displacement, at his own discretion, each subject sprinted forward to cone B and touched the top of the cone with the right hand; BC displacement, facing forward the participant shuffled to the left to cone C and touched the top of the cone with the left hand; CD displacement, the soccer player then shuffled to the right to cone D and touched its top; DB displacement, the players shuffled back to the left to cone B and touched its top; BA displacement, the players moved backward as quickly as possible and returned to line A. Players performed two maximal trials, with 1-minute of rest in between. The best value achieved was selected for analysis.

### Linear 30-m Sprint Test

For maximal sprinting physical fitness assessment, athletes completed a S-30 sprint. The S-30 involved single maximal-effort sprints from a stationary start. Players initiated the sprint at their own discretion, positioning the foot 0.5-m behind the start line. Thirty-meter linear sprint performance was assessed using a double-beam photocell system (Witty, Microgate^®^, Italy). Athletes performed two maximal trials, with 1-minute of rest in between. The best value achieved was selected for analysis.

### Repeated Sprint With COD Test

In addition to the linear sprint, athletes completed a RSCOD test. The RSCOD test included 6 sprints, with a passive recovery period of 20-seconds in between (Okuno et al., 2013). Each sprint involved 15-m of linear sprint, a COD of 180°, and another 15-m linear sprint [15-m + 15-m (COD-180°)]. Times were recorded with a double-beam photocell system (Witty, Microgate^®^, Italy). Athletes received verbal encouragement during the test. The players were verbally and visually informed to assume the starting position 0.5-m behind the starting line 6-seconds before each sprint. Also a 3-seconds countdown was visually provided with a light panel (Microgate^®^, Italy) that informed of the start of the next sprint. The fastest sprint (RSCODb) and the total time of all sprints (RSCODt) were retained for further analyses.

### Warm-Up Protocols

**FIGURE 2:**
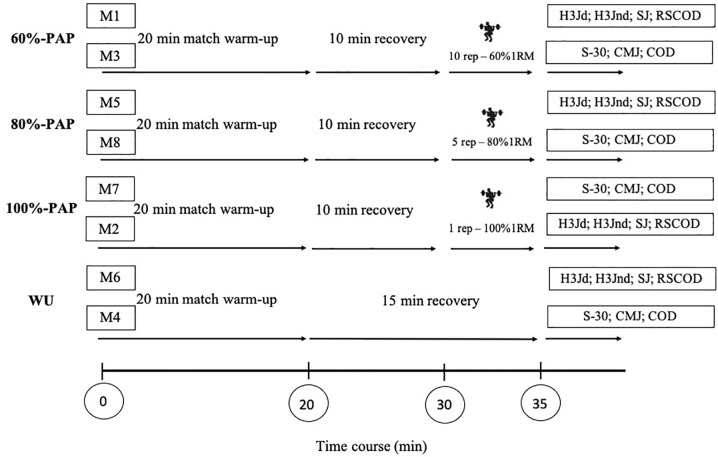
Overview of the cross-over experimental design, indicating the control and three different warm-up conditions. The 15-minutes recovery period was included during control condition. The 10-minutes plus 5-minutes recovery period were included during PAP protocols, to simulate match conditions. The tests were divided at two measurement (M) day corresponding to each protocols. 60%-1RM, 80%-1RM, and 100%-1RM refer to match warm-up post-activation potentiation (PAP) protocols at 60, 80 and 100% of 1RM, respectively; WU, match warm-up protocol; M, measurement; H3Jd, triple hop test with dominant leg; H3Jnd, triple hop test with non-dominant leg; SJ, squat jump test; RSCOD, repeated sprint with change of direction test; S-30, linear 30-m sprint test; CMJ, countermovement jump test; COD, change of direction ability test.

The overview of warm-up protocols is presented in Figure 2. A control warm-up protocol (WU) was designed according to athlete’s regular warm-up practices. The WU included 7-minutes of general warm-up [i.e., continuous moderate-intensity (≤10 km/h^−1^) running; general main-joint movements (mobility of upper and lower extremities; proprioceptive drills [landings with dominant and non-dominant leg after low-intensity frontal and lateral jumps)], 3-minutes of specific warm-up [i.e., elastic-bands resisted drills and ballistic drills (lateral and frontal movements on 5-m and simulations of ball shots wearing an elastic on the ankles or knees)], 5-minutes of ball drills (i.e., basic technical drills with a partner), and 5-minutes of small-sided games (i.e., 5vs5 in a 30 × 20-m size pitch). After the WU athletes rested passively for 15-minutes before testing, as it happens in a competition match. The experimental PAP warm-ups included the same exercises as the WU, with the addition (10-minutes after the end of WU) of one set of half back squats at 60, 80, or 100% of 1RM for a total of 10 repetitions (60%-1RM), 5 repetitions (80%-1RM), or 1 repetition (100%-1RM), respectively. All repetitions were performed at maximal voluntary concentric velocity. After each PAP load, athletes rested passively for 5-minutes before the H3JD, H3Jnd (measure in day 1) and S-30 tests (measure in day 2), 6-minutes before the SJ (measure in day 1) and CMJ tests (measure in day 2), and 8-minutes before the RSCOD (measure in day 1) and COD (measure in day 2), respectively.

### Statistical Analyses

Data is presented as mean ± standard deviation (SD). All data were first log-transformed to reduce biases arising from non-uniformity error. The standardized difference or effect size [ES, 90% confidence limits (CL)] in the selected variables was calculated. Threshold values for Cohen’s *ES* statistics were >0.2 (small), >0.6 (moderate), and >1.2 (large) (Hopkins et al., 2009). For within-group comparisons, the chances that the differences in performance were better/greater [i.e., greater than the smallest worthwhile change (0.2 multiplied by the between-subject standard deviation, based on Cohen’s *d* principle)], similar, or worse/smaller were calculated. Quantitative chances (QC) of beneficial/better, similar/trivial, or detrimental/poorer effect were assessed qualitatively as follows: <1%, almost certainly not; >1–5%, very unlikely; >5–25%, unlikely; >25–75%, possible; >75–95%, likely; >95–99%, very likely; and >99%, most likely (Hopkins et al., 2009). If the chance that the true value is >25% beneficial and >0.5% chance that it is harmful, the clinically effect was considered as unclear. However, the clinical inference was declared as beneficial when odds ratio of benefit/harm was >66% (Hopkins et al., 2009). A specific Excel spreadsheet from sportsci.org was used to examine the within-group (between PAP protocols) (xPostOnlyCrossover.xls) comparisons.

## Results

The mean 1RM half-squat for this study was 114.3 ± 15.0 kg. Table 1 shows the results for each test after each warm-up protocol.

**Table 1:** Physical performance of soccer players after different warm-up protocols.

	WU	100%-1RM	80%-1RM	60%-1RM
H3Jd (m)	6.53 ± 0.34	6.40 ± 0.36	6.73 ± 0.41	6.26 ± 0.49
H3Jnd (m)	6.57 ± 0.26	6.56 ± 0.43	6.71 ± 0.36	6.32 ± 0.39
SJ (cm)	37.4 ± 4.9	36.7 ± 4.9	38.8 ± 4.7	34.5 ± 5.4
CMJ (cm)	39.8 ± 3.2	38.6 ± 5.3	40.7 ± 4.7	38.4 ± 3.2
COD (s)	7.23 ± 0.27	7.39 ± 0.38	7.12 ± 0.26	7.38 ± 0.33
RSCODb (s)	5.77 ± 0.15	5.94 ± 0.11	5.67 ± 0.13	5.89 ± 0.21
RSCODt (s)	35.7 ± 0.65	35.8 ± 0.62	35.0 ± 0.67	35.5 ± 0.70
S-30 (s)	4.05 ± 0.23	4.13 ± 0.23	4.09 ± 0.28	4.04 ± 0.23

*H3Jd and H3Jnd, triple hop test with dominant and non-dominant leg, respectively, SJ, squat jump; CMJ, countermovement jump; COD, change of direction ability test; RSCODb and RSCODt, repeated sprint with change of direction best and total times, respectively, S-30, linear 30-m sprint test. WU, control warm-up protocol; 100%-1RM, 80%-1RM, and 60%-1RM, PAP warm-up protocols at 60, 80, and 100% of one repetition maximum in half-squat.*

Comparisons between the control warm-up and the PAP warm-ups are indicated in Table 2. Likely to most likely improvements were shown in H3Jd, H3Jnd, COD, RSCODb, and RSCODt only after the 80%-1RM protocol in comparison to WU. Furthermore, a possibly greater enhancement was found in RSCODt after the 60%-1RM protocol compared to WU. Conversely, 100%-1RM and 60%-1RM protocols induced possible to most likely poorer performance in the H3Jd, H3Jnd, SJ, CMJ, COD, and RSCODb in comparison to WU. Similarly, a likely poorer S-30 performance was observed after 100%-1RM compared to WU.

**Table 2:** Comparisons of soccer player’s physical performance after a traditional (control) warm-up versus three different warm-up conditions.

	% (CL90%)	ES (CL90%)	Chances	Outcome
**WU vs. 100%-1RM**				
H3Jd	−2.0 (−4.4; 0.5)	−0.35 (−0.79; 0.08)	2/24/73%	Possibly
H3Jnd	−0.3 (−2.7; 2.2)	−0.07 (−0.65; 0.52)	21/44/34%	Possibly
SJ	−2.0 (−8.1; 4.5)	−0.15 (−0.61; 0.32)	10/48/42%	Possibly
CMJ	−3.5 (−8.7; 1.9)	−0.41 (−1.04; 0.21)	5/22/73%	Possibly
COD	−2.1 (−4.4; 0.2)	−0.50 (−1.04; 0.04)	2/14/83%	Likely
RSCODb	−3.0 (−3.9; −2.1)	−1.03 (−1.32; −0.73)	0/0/100%	Most Likely
RSCODt	−0.3 (−0.5; −0.1)	−0.14 (−0.25; −0.03)	0/83/17%	Likely trivial
S-30	−1.7 (−2.2; −1.2)	−0.28 (−0.36; −0.20)	0/5/95%	Likely
**WU vs. 80%-1RM**				
H3Jd	3.0 (0.7; 5.3)	0.52 (0.13; 0.91)	92/8/0%	Likely
H3Jnd	2.2 (0.2; 4.2)	0.51 (0.05; 0.97)	88/11/1%	Likely
SJ	3.8 (−3.0; 11.1)	0.27 (−0.22; 0.76)	60/34/6%	Unclear
CMJ	2.0 (−2.6; 6.7)	0.22 (−0.30; 0.75)	53/38/9%	Unclear
COD	1.6 (−0.4; 3.5)	0.38 (−0.09; 0.86)	75/22/2%	Likely
RSCODb	1.6 (0.5; 2.8)	0.58 (0.16; 1.00)	93/6/0%	Likely
RSCODt	2.0 (1.2; 2.7)	0.99 (0.60; 1.38)	100/0/0%	Most Likely
S-30	−0.9 (−3.7; 1.7)	−0.15 (−0.60; 0.29)	9/49/42%	Unclear
**WU vs. 60%-1RM**				
H3Jd	−4.3 (−5.8; −2.7)	−0.77 (−1.06; −0.48)	0/0/100%	Most Likely
H3Jnd	−3.8 (−6.0; −1.6)	−0.91 (−1.46; −0.37)	0/2/98%	Very Likely
SJ	−8.0 (−11.8; −4.0)	−0.60 (−0.91; −0.30)	0/2/98%	Very Likely
CMJ	−3.5 (−5.0; −1.9)	−0.40 (−0.59; −0.22)	0/3/97%	Very Likely
COD	−2.0 (−3.3; −0.7)	−0.48 (−0.80; −0.17)	0/6/93%	Likely
RSCODb	−2.1 (−3.7; −0.4)	−0.72 (−1.28; −0.15)	1/6/94%	Likely
RSCODt	0.5 (−0.1; 1.1)	0.26 (−0.03; 0.56)	65/34/1%	Possibly
S-30	0.2 (0.1; 0.4)	0.04 (0.01; 0.07)	0/100/0%	Most Likely trivial

*To avoid a misinterpretation of the results, positive results indicate a better performance in favor of the PAP warm-up protocol, while negative results show a better performance in favor of the control warm-up protocol. CL, confidence limits; ES, effect size; PAP, post-activation potentiation; H3Jd and H3Jnd, triple hop test with dominant and non-dominant leg, respectively; SJ, squat jump; CMJ, countermovement jump; COD, change of direction ability test; RSCODb and RSCODt, repeated sprint with change of direction best and total times, respectively; S-30, linear 30-m sprint test. WU, Control warm-up protocol; 100%-1RM, 80%-1RM, and 60%-1RM, post-activation potentiation warm-up protocols at 60, 80, and 100% of one repetition maximum in half-squat.*

Comparisons between the PAP warm-up protocols are indicated in Table 3. Possibly to most likely improvements were shown in H3Jd, H3Jnd, SJ, CMJ, COD, RSCODb, and RSCODt after the 80%-1RM warm-up protocol in comparison to the 100%-1RM and 60%-1RM warm-up protocols. In addition, a possible better performance was achieved in the S-30 after the 80%-1RM warm-up compared to the 100%-1RM warm-up, while the 60%-1RM warm-up induced possible and very likely better S-30 performance in comparison to the 80%-1RM and 100%-1RM warm-up protocols, respectively. Finally, better performance in H3Jd (possible), H3Jnd (likely) and SJ (likely) were observed after 100%-1RM warm-up compared to 60%-1RM warm-up, whereas better performance in RSCODb (possible), RSCODt (likely), and S-30 (very likely) were observed after the 60%-1RM warm-up compared to the 100%-1RM warm-up.

**Table 3:** Comparisons of soccer player’s physical performance after three different warm-up conditions.

	% (CL90%)	ES (CL90%)	Chances	Outcome
**100%-1RM vs. 80%-1RM**				
H3Jd	5.1 (3.7; 6.5)	0.79 (0.58; 1.00)	100/0/0%	Most Likely
H3Jnd	2.5 (1.1; 3.9)	0.35 (0.15; 0.55)	90/10/0%	Likely
SJ	5.9 (3.3; 8.7)	0.40 (0.22; 0.57)	96/4/0%	Very Likely
CMJ	5.7 (2.5; 9.1)	0.38 (0.16; 0.59)	92/8/0%	Likely
COD	3.6 (1.4; 5.7)	0.65 (0.25; 1.04)	97/3/0%	Very Likely
RSCODb	4.5 (3.6; 5.4)	2.15 (1.70; 2.59)	100/0/0%	Most Likely
RSCODt	2.3 (1.6; 2.9)	1.21 (0.84; 1.57)	100/0/0%	Most Likely
S-30	0.8 (−2.1; 3.5)	0.13 (−0.36; 0.63)	41/47/12%	Possibly
**100%-1RM vs. 60%-1RM**				
H3Jd	−2.3 (−5.6; 1.1)	−0.37 (−0.92; 0.17)	4/24/71%	Possibly
H3Jnd	−3.5 (−6.8; −0.1)	−0.51 (−1.01; −0.01)	1/13/86%	Likely
SJ	−6.1 (−11.8; 0.0)	−0.43 (−0.86; 0.00)	1/16/83%	Likely
CMJ	0.1 (−5.7; 6.2)	0.01 (−0.39; 0.40)	20/62/18%	Possibly trivial
COD	0.1 (−2.4; 2.5)	0.01 (−0.43; 0.46)	23/57/20%	Possibly trivial
RSCODb	0.9 (−0.9; 2.7)	0.41 (−0.44; 1.26)	67/22/11%	Possibly
RSCODt	0.8 (0.2; 1.5)	0.43 (0.08; 0.78)	87/12/0%	Likely
S-30	1.9 (1.4; 2.4)	0.34 (0.25; 0.43)	99/1/0%	Very Likely
**60%-1RM vs. 80%-1RM**				
H3Jd	7.6 (4.3; 10.9)	0.88 (0.51; 1.24)	100/0/0%	Most Likely
H3Jnd	6.2 (3.1; 9.4)	0.90 (0.45; 1.34)	99/1/0%	Very Likely
SJ	12.9 (5.8; 20.3)	0.72 (0.34; 1.11)	98/2/0%	Very Likely
CMJ	5.6 (0.3; 11.2)	0.61 (0.04; 1.18)	89/10/1%	Likely
COD	3.6 (1.5; 5.9)	0.87 (0.36; 1.39)	98/2/0%	Very Likely
RSCODb	3.8 (1.6; 6.0)	1.46 (0.64; 2.29)	99/1/0%	Very Likely
RSCODt	1.5 (0.6; 2.4)	0.69 (0.27; 1.10)	97/3/0%	Very Likely
S-30	−1.2 (−3.8; 1.6)	−0.16 (−0.53; 0.21)	5/52/42%	Possibly

*To avoid a misinterpretation of the results, positive results denote a better performance in favor of the PAP warm-up protocol indicated at the right, while negative results denote a better performance in favor of the PAP warm-up protocol indicated at the left. CL, confidence limits; ES, effect size; PAP, post-activation potentiation; H3Jd and H3Jnd, triple hop test with dominant and non-dominant leg, respectively; SJ, squat jump; CMJ, countermovement jump; COD, change of direction ability test; RSCODb and RSCODt, repeated sprint with change of direction best and total times, respectively; S-30, linear 30-m sprint test. WU, control warm-up protocol; 100%-1RM, 80%-1RM, and 60%-1RM, PAP warm-up protocols at 60, 80, and 100% of one repetition maximum in half-squat.*

## Discussion

The aim of this study was to compare the effects of different conditioning intensities on the physical fitness (i.e., PAP) of professional male field soccer players. Main results indicate improvements in jumping, single and repeated COD speed after the 80%-1RM protocol in comparison to WU. Moreover, better jumping, as well as single and repeated COD speed improvements were observed after the 80%-1RM compared to the 60%-1RM and 100%-1RM protocols. Therefore, a moderate intensity (i.e., 80%-1RM) appears to be more effective than low (i.e., 60%-1RM) and maximal (i.e., 100%-1RM) warm-up strategies to induce greater PAP, including greater jumping, single and repeated COD speed in male soccer players.

Regarding jumping performance, likely to most likely improvements were shown in H3Jd and H3Jnd only after the 80%-1RM protocol in comparison to WU. Moreover, possibly to most likely improvements were shown in H3Jd, H3Jnd, SJ, and CMJ test after the 80%-1RM warm-up protocol in comparison to the 100%-1RM and 60%-1RM warm-up protocols. Improvements in jumping performance after loaded squat PAP protocols have been previously observed in male athletes from team-sports such as rugby, volleyball and soccer (Gouvêa et al., 2013), and may be explained by several neuro-mechanical short-term adaptations (e.g., increased muscle-tendon stiffness) (Tillin and Cooke, 2009). Moreover, the PAP effects depend on the balance between fatigue and neuromuscular potentiation (Tillin and Bishop, 2009), which in turn depends on the load-related intensity used (Sale, 2004). In the current study, a load of moderate-intensity (i.e., 80%-1RM) induced greater jumping performance improvements compared with loads of lower (i.e., 60%-1RM) or greater (i.e., 100%-1RM) intensity, agreeing with previous studies that found greater PAP effects after loads of intermediate intensity (Gouvêa et al., 2013). Of note, the greater PAP effect after intermediate-intensity loads may be particularly important when PAP actions are performed with the intention of maximizing movement velocity, leading to the recruitment of fast-twitch muscle fibers, which is considered a key factor to induce PAP (Turner et al., 2015). This improvement in jumping performance after loaded squats have been observed even after 6 h from the PAP warm-up (Saez-Saez de Villarreal et al., 2007). In addition, is important to note that soccer players’ characteristics may affect the PAP magnitude (Sanchez-Sanchez et al., 2018), thus current results should be interpreted considering the high training level of the soccer players.

The results indicate likely improvements in the COD after the 80%-1RM protocol in comparison to WU, and likely poorer performance after the 100%-1RM and 60%-1RM protocols. Current outcomes are difficult to compare with previous findings, given the limited literature related to COD performance and PAP (Lockie et al., 2017). However, two previous studies observed improvements in COD performance after warm-up actions that included loaded exercises (Zois et al., 2011; Sole et al., 2013), and the improvement may be related to acute increase of reactive strength (Sole et al., 2013). Reactive strength is the ability to quickly change from the eccentric to the concentric phase during a stretch-shortening cycle muscle action (Young et al., 1998). In this sense, a greater reactive strength may help to improve the ability to perform sudden stops and to accelerate from there (Spiteri et al., 2013), hence improving COD speed (Sheppard and Young, 2006). Of note, a greater PAP effect (i.e., greater COD performance) was observed after the 80%-1RM versus the 60%-1RM, since the load used in the 80%-1RM may help to maximize the acceleration phase of the COD action (McBride et al., 2005), implicating a better use of the stretch-shortening cycle in the deceleration-acceleration transition of the COD movement. However, the 100%-1RM did not maximize COD performance. In this sense, the PAP effect may not proportionally depend on the load used (i.e., the higher the better), but other factors also may modulate the effect, such as the muscle fiber type, athletes’ performance level, exercise type, time interval between the conditioning stimulus and the performance testing, among others (Sanchez-Sanchez et al., 2018). In fact, it has been suggested that there are PAP responders that may benefit from exercises designed to induce PAP, whereas others may not respond (Evetovich et al., 2015).

Regarding the RSCOD test, likely and most likely improvements were found in RSCODb and RSCODt with 80%-1RM in comparison to WU, respectively. Improvements in RSCOD performance after loaded back half-squat PAP protocols have been previously observed in elite male handball (Okuno et al., 2013) and soccer players (Sanchez-Sanchez et al., 2018). The 80%-1RM may improve neuromuscular capacity (Hodgson et al., 2005), allowing an increase in athlete’s power (Tillin and Bishop, 2009), thus better ability to repeat sprints (Glaister, 2005). Although warm-ups delivered to induce PAP may increase RSCOD performance, the load used must be applied with caution. Heavy loads (>90% 1RM), with recovery times of 8-minutes, may allow improvements in the total time and sprint time in a repeated sprint test (Low et al., 2015). However, the use of a heavier load (i.e., 100%-1RM protocol) may induce most likely poorer performance in the RSCODb in comparison to a WU, even when 8-minutes of rest are allowed. Although potentiation and fatigue coexist, the 100%-1RM protocol may have induced fatigue to a greater extent than its PAP effect, potentially due to decreased release of calcium from the sarcoplasmic reticulum, leading to reduced calcium concentration in the myoplasm (Rassier and MacIntosh, 2000). In this sense, to optimally induce PAP, the load used must be selected accurately in male soccer players (Hammami et al., 2018). Although previous studies analyzed the effect of PAP warm-ups on RSCOD (Sanchez-Sanchez et al., 2018), this is the first study that compared the effects of three different loaded protocols on RSCOD performance, a key fitness specific-trait for soccer (Schimpchen et al., 2015), which determines match physical performance (Rampinini et al., 2007) and differentiates between competitive levels (Rampinini et al., 2009). Therefore, current findings may help practitioners to optimally prepare players before a match.

No changes in S-30 performance was observed after the 60%-1RM and the 80%-1RM compared to WU. No effects in sprint time after heavy loaded squat PAP protocols have been previously observed in soccer players (Tillin and Cooke, 2009). In contrast, the positive effects of heavy-load squats was obtained at distances of 10 to 40-m (McBride et al., 2005; Chatzopoulos et al., 2007; Rahimi, 2014). The mechanisms that underlie the effects of PAP warm-ups using loaded exercises on sprint performance have not been clarified (McBride et al., 2005). Maximal sprint velocity (i.e., distances >30 m) may depend on the force of the extensor muscles of the hip in order to re-incorporate the leg in the swing phase and thus maintain an adequate stride length (Weyand et al., 2000). Although the muscle force may be increased through a PAP warm-up, inducing an increased muscle stiffness (Moir et al., 2009), based on previous research, it was speculated that the PAP should be related to the volume of the pre-load (Evetovich et al., 2015). The PAP has traditionally been induced through the use of multiple sets of heavy isotonic resistance exercise (Wilson et al., 2013), but in this study only one set of 60%-1RM and 80%-1RM was applied. From a practical perspective, given the timeframe separating the end of the warm-up and the start of a soccer match, there is no time to include multiple sets (Russell et al., 2014). On the other hand, a likely poorer performance after 100%-1RM compared to WU was observed. It is possible that the recovery time used in the present study (i.e., 5-minutes) was not enough to elicit a significant PAP effect after the 100%-1RM in some players. Considering that fatigue and potentiation co-exist (Rassier and MacIntosh, 2000), shorter rest intervals may increase fatigue (Tillin and Cooke, 2009). In this sense, <4 min of rest after a PAP warm-up of 5-RM did not induce an increase in sprint performance (Tillin and Cooke, 2009). However, 5 min of rest after a PAP warm-up, as used in the current study, seems to be adequate in order to reload phosphocreatine stores (Chatzopoulos et al., 2007). Still, this did not explain the lack of improvements or even poorer sprinting capability after the 100%-1RM strategy used in the current study. It has been suggested that in order to improve sprinting performance, the potential effect of heavier PAP protocols, including load scheme and rest time, should be prescribed on an individual basis (Mola et al., 2014).

Using an 80%-1RM protocol could be an effective strategy to enhance the physical performance of elite male soccer player, with potential implications for a better performance, especially at the beginning of games. Coaches should carefully consider the recovery time between the PAP application and the start of a match in order to reduce the risk of fatigue. Moreover, although in the current study an 80%-1RM protocol induced greater mean improvements in the physical performance of soccer players, PAP protocols should be elaborated considering athlete’s individual characteristics.

Potential limitations of the current study are related to the lack of physiological and biochemical measurements, in order to further understand the underlying factors related with the observed PAP phenomenon. The limited number of subjects involved in the current study could be recognized as an additional limitation, in line with the use of magnitude-based inferences (MBI). Such statistical approach has been criticized as may induce a greater risk of type I error (Sainani, 2018). On the other side, its use have been strongly supported in sport science studies (Batterham and Hopkins, 2019). Future studies should strive to elucidate if current results may be transferred to competition scenarios, analyzing performance indices during a match, such as covered distance at high-intensity, accelerations, among others short-term high-intensity actions. In addition, future studies may analyse the potential interfering effect (if any) of repeated physical fitness test (i.e., H3JD, SJ) performed on the same testing session and its role on PAP after warm-ups using different 1RM back-squat intensities.

## Conclusion

In conclusion, a moderate intensity (i.e., 80%-1RM back squat) may induce greater improvements (i.e., PAP effect) in jumping, repeated COD speed and non-repeated COD speed in elite level male soccer players when compared to low (60%-1RM) and high (100%-1RM) intensity warm-up protocols.

## Ethics Statement

This study was carried out in accordance with the recommendations of “Ethics Committee, Pontifical University of Salamanca” with written informed consent from all subjects. All subjects gave written informed consent in accordance with the Declaration of Helsinki. The protocol was approved by the “Ethics Committee, Pontifical University of Salamanca (Annex III, Act 13/2/2019).”

## Author Contributions

CP, RR-C, and JS-S designed the work. CP, DH, and JS-S acquired the data. CP, FN, RR-C, JS-S, and OG-S analyzed and interpreted of data. All authors drafted the manuscript, critically revised the manuscript and approved the final version of the manuscript to be published. CP, RR-C, FN, and JS-S agreed to be accountable for all aspects of the work in ensuring that questions related to the accuracy or integrity of any part of the work were appropriately investigated and resolved.

## Conflict of Interest Statement

The authors declare that the research was conducted in the absence of any commercial or financial relationships that could be construed as a potential conflict of interest.
